# Hernia Basecamp—A Free to Use, Online Hernia Learning Platform. Analysis of Its Use Since Launch in June 2021

**DOI:** 10.3389/jaws.2023.11803

**Published:** 2023-09-29

**Authors:** A. de Beaux, S. Becker, T. Parent, G. Temporal, R. Kummer, C. Allouard, B. East

**Affiliations:** ^1^ Spire Murrayfield Hospital, Edinburgh, United Kingdom; ^2^ WebSurg, The Online University of IRCAD, Research Institute Against Digestive Cancer (IRCAD), Strasbourg, France; ^3^ Medtronic International Trading Sarl, Tolochenaz, Switzerland; ^4^ Medical Affairs, Medtronic, Paris, France; ^5^ 3rd Department of Surgery, 1st Medical Faculty of Charles University at Motol University Hospital, Prague, Czechia

**Keywords:** Hernia Basecamp, UEMS, education, WebSurg, continuing medical education

## Abstract

**Introduction:** Hernia Basecamp is an online learning platform hosted within the WebSurg website. One of the drivers of its development was to cover the syllabus of the UEMS AWS examination, but it is a learning resource in its own right. There are currently 205 video lectures, with a number of them selected to create 10 modules of 3 h each with UEMS CME accreditation. The aim of this study was to review the Hernia Basecamp usage since launch in June 2021.

**Methods:** The Hernia Basecamp WebSurg platform was interrogated using Matomo Analytics in January 2023 (19 month period since launch). Data on the number of visits, pages looked at and time spent on the platform per visit, along with the number of CME modules taken and passed were collected.

**Results:** Users from 146 countries visited the Hernia Basecamp site 17,171 times (6,586 times, 38.4% in first 9 months). The top 5 countries by visitors were the United Kingdom, Mexico, Spain, United States and Germany (accounting for 29.4% of the visits). The average time spent per visit was 11 min 37 s (range: 47 s–49 min 4 s), and the number of pages/videos viewed per visit was 8.1 (range: 2–21). The number of UEMS CME modules taken was 675, and 326 (48%) of these tests were passed.

**Conclusion:** In the first 19 months from launch, Hernia Basecamp provided over 3,000 h of hernia education. The UEMS approved CME accreditation tests were commonly used.

## Introduction

Following the realisation of the global impact of hernia surgery, the European Union of Medical Specialists (UEMS) recognised abdominal wall surgery as a separate subspecialty of general surgery in 2019.^1^ A working group called the Abdominal Wall Section (AWS) was established, defining an “Abdominal Wall Surgeon,” and drawing up a syllabus of knowledge and skills expected of such a surgeon. Working with the European Hernia Society (EHS), through the presence of a secretary for UEMS who was added to the EHS Board in 2020, the UEMS AWS developed the examination for the conferring of Fellow of the European Board of Surgery, Abdominal Wall Section, FEBS AWS for short. This examination is currently held before the Annual EHS Congress, the first that was held at Copenhagen, Denmark in 2021, the second one in Manchester, United Kingdom in 2023 and the last one in Barcelona Spain in 2023. Further examinations are planned in Prague (Czech Republic), Paris (France), and Porto (Portugal) in 2024, 2025, and 2026, respectively.

In the midst of the COVID-19 pandemic, and on the back of a successful online learning platform for gynaecological surgery hosted on WebSurg,^2^ the online learning university portal of the French Strasbourg-based IRCAD Institute, a number of the authors came up with the plan to set up an online, free to use, video lecture-based learning platform to cover the UEMS knowledge syllabus. It was called “Hernia Basecamp”.^3^ At its launch in June 2021, over 200 video lectures, and recently published EHS guidelines were grouped into eight topic areas. A number of these lectures and guidelines were selected to create 10 e-learning modules (ELMs) of 3 h maximum for each, with a 10-question multiple choice questionnaire (MCQ) at the end of each module (8 out of 10 correct questions pass rate). UEMS CME accreditation was applied for and granted for all 10 ELMs.

The aim of this study was to review Hernia Basecamp usage and the CME module test results since its launch in June 2021.

## Methods

The Hernia Basecamp WebSurg platform^3^ was interrogated using Matomo Analytics in January 2023 (over a 19-month period since launch). Data on the number of visits, pages looked at, and time spent on the platform per visit, along with the number of CME-accredited ELMs were collected. Data was also collected on the countries and the world regions accessing the site. Data on the number of CME module tests taken, the pass rate, and the country of the examinee were collected.

## Results

Over the 19 months period since launch, users from 146 countries visited the Hernia Basecamp site 17,171 times (6,586 times, 38.4% in the first 9 months). The top 5 countries by visitors included the United Kingdom, Mexico, Spain, United States, and Germany (accounting for 29.4% of the visits). Due to changes in the analytics of the Hernia Basecamp, detailed breakdown in these visits by region, pages opened, time spent on the site, and the bounce rate could only be calculated from April 2022 (i.e., over a 10 months period) and these are shown in Table 1.

**TABLE 1:** Number of visits to the Hernia Basecamp platform, actions per visit, time per visit, and the bounce rate from the platform by world region over a 10 months period (April 2022–January 2023).

Continent	Visits	Actions	Actions per visit Mean (range)	Time on website at visit Mean (range)	Bounce rate (%)
Europe	6,209	48,797	7.9 (2–21)	11 min 29 s (47 s–49 min)	27
Asia	1,373	12,221	8.9 (2–51)	12 min 40 s (21 s–1 h 11 min)	20
North America	1,111	7,498	6.7 (2–37)	9 min 37 s (39 s–2 h 6 min)	29
South America	1,016	9,364	9.2 (2–64)	13 min 23 s (23 s–1 h 49 min)	13
Africa	397	4,247	10.7 (2–68)	16 min 40 s (45 s–2 h 41 min)	17
Central America	241	1,667	6.9 (2–52)	11 min 18 s (30 s–1 h 31 min)	25
Oceania	238	1,217	5.1 (2–66)	7 min 41 s (41 s–1 h 50 min)<	34
**Total**	**10,585**	**85,011**	**8.1 (2–21)**	**11** **min 37** **s (39** **s–2** **h 41** **min)**	

The total number of UEMS CME-accredited ELMs taken was 675. The number of tests taken, the number of passes, and the pass rate per module are shown in Table 2. The United Kingdom, Switzerland, and Germany were the top 3 countries representing 36% of the ELMs passed.

**TABLE 2:** The number of EUMS CME-accredited ELM tests taken, the number of passes, and the pass rate per module (June 2021 to January 2023).

CME ELM	Taken	Passed (%)
Groin anatomy, preoperative and postoperative management of groin hernia	215	23 (11)
Groin hernia therapy	78	59 (76)
Incisional hernia: anatomy, preoperative and postoperative recommendations	31	27 (87)
Incisional hernia: minimally invasive therapy	26	23 (88)
Incisional hernia: open therapy	25	20 (80)
Inguinal hernia guidelines	108	61 (56)
Parastomal hernia	48	40 (83)
Primary ventral: anatomy, preoperative and postoperative recommendations	59	48 (81)
Primary ventral hernia: guidelines	53	9 (17)
Primary ventral hernia: therapy	32	16 (50)
**Total**	**675**	**326 (48)**

## Discussion

The online, free to use, video lecture-based, and EHS guideline Hernia Basecamp learning platform has provided over 3,000 h of abdominal wall surgery education, reaching most parts of the globe. This was a much bigger impact and reach of the platform than anticipated at the initiation of the program. The original concept was to provide the flesh to the EUMS knowledge curriculum, but as the platform emerged, it became evident that it was an abdominal wall surgery education tool in its own right. Many of the visitors will not take the FEBS AWS examination, but the ability to learn and have recognition of the learning with UEMS-accredited ELMs. This includes a novel concept of being able to demonstrate knowledge around recently published EHS guidelines with UEMS CME accreditation.

The need for ongoing hernia education is evident. Until about 30 years ago, a relatively small number of open non-mesh operations were available covering the hernia repair spectrum. However, there has been a surge in techniques, including laparoscopic and robot-assisted techniques, along with exploiting different layers in the abdominal wall, new mesh science, fixation devices, and so on. Indeed, the learning needs to train a hernia surgeon and also keep them up-to-date has been recognised as a somewhat mammoth task [1]. Hence the recognition by the UEMS of the need to recognise hernia and abdominal wall repair as a subspecialty of general surgery in its own right.

Other online hernia learning platforms exist. The EHS has been running the Fundamentals in Abdominal Wall Surgery for several years. It is based around 12 h of lectures in three sessions, with MCQ self-study tests at the end of each module, although there is no accreditation of these tests by a regulatory body. Hernia U^4^ is another online website running regular live operating and webinar series, along with a lecture series. A recent article seeking feedback about the Hernia U program identified that the majority of users had changed their hernia practice because of new information they learnt from the platform [2].

It is evident that some visitors land on the website, and leave again in a few seconds, the so-called bounce rate. This is common on any website, although the characteristics of these “bouncing” visitors is not known. However, this should not take away from the importance of the platform, and those who are using it regularly, on some occasions over 2 h at a time. We note that while most visitors are in Europe, this is to be expected as the promotion and indication for the platform to begin with was Eurocentric.

It was hoped that the UEMS CME-approved ELMs would be popular, as the process for UEMS accreditation is a time-consuming process. And we feel it has been with nearly 700 tests taken over the analysis period. The results of these tests have been generally encouraging, with a high pass rate in most modules. However, as seen in Table 2, basic knowledge in groin hernia is somewhat lacking, and knowledge around guidelines is also disappointingly low. It is possible that the modules are not fit for purpose, but this is unlikely as the answer to every MCQ question is presented clearly somewhere in the core lectures that make up the module. From anecdotal evidence, some candidates are taking the MCQ examination without watching all ELMs and are subsequently failing as their knowledge deficit is exposed. As per the UEMS rules, a candidate is locked out of the MCQ post-test quiz for a specific module for a year after failing the test (80% of correct answers are required to pass each test). Hernia surgeons should read guidelines, and the EHS-produced guidelines continue to evolve and seek to use best guideline methodology. The ability to read the guidelines, and then pass UEMS CME-accredited evidence of this is a powerful incentive for many surgeons who are required to produce evidence of ongoing learning.

No learning platform can stand still and needs regular work to revise and update lectures as new evidence emerges, in addition to cover new material. Interpretation of the scientific literature remains an essential skill for surgeons wishing to perfect and develop their skills and knowledge throughout their career. A module on this area is necessary and is one of the expansion projects planned. The strategic partnership between the EHS and the British Journal of Surgery Society is noted, and the work on the BJS Academy website^5^ in this area is area is welcomed to help educate surgeons in this important topic. Readers of this article, and users of the Hernia Basecamp platform are encouraged to offer suggestions for improvement, revision, and indeed contribute new lectures and CME modules as they see necessary.

The major sponsors of WebSurg, the online university of IRCAD, are industry partners. And Medtronic, in particular, have funded the Hernia Basecamp set-up and running cost through their unrestricted educational sponsorship. Funding is provided free of any attempt to influence the lectures, subjects for discussion, content, and choice of faculty members. Of note, the content and thus intellectual rights of each lecture belongs to the person creating the lecture. They are free to make their own lecture, disclosing any conflicts of interest or financial ties. It must be underlined that templates about topics to cover within a lecture were provided from the surgical team (BE/AdeB), with a strong emphasis on evidence-based medicine where evidence exists.

There are limitations to the study. The changes in the analytics of the Hernia Basecamp platform since launch resulted in some of the study parameters being unavailable for analysis over the first 9 months since launch. In addition, knowledge about the users is not known. They are assumed to be all surgeons of various experience. While the content of Hernia Basecamp is primarily around knowledge, it is hoped that such knowledge would improve hernia practice, and indeed improve patient outcomes, by those who use the platform. These are areas for future research.

In conclusion, Hernia Basecamp has realised its early goals, rapidly becoming a hernia related learning source used by surgeons world wide. Keeping the content up-to-date, relevant and interesting is an ongoing task. Furthermore, the success of this platform has encouraged other groups to set up other basecamps [3].

## Data Availability

The raw data supporting the conclusion of this article will be made available by the authors, without undue reservation.

## Footnotes

1
https://uemssurg.org/surgicalspecialties/abdominal-wall-surgery/

2
https://websurg.com/en/dashboard/

3
https://websurg.com/en/basecamp/hernia

4
https://www.herniau.com/

5
www.bjssociety.com
